# Supplementary figures and images for: Japanese encephalitis virus activates the NLRP3/caspase-1/GSDMD signaling pathway in dopaminergic neurons
Source: PLoS Negl Trop Dis. 2026 Jan 22;20(1):e0013924. doi: 10.1371/journal.pntd.0013924 (PMC12893654; doi:10.1371/journal.pntd.0013924)

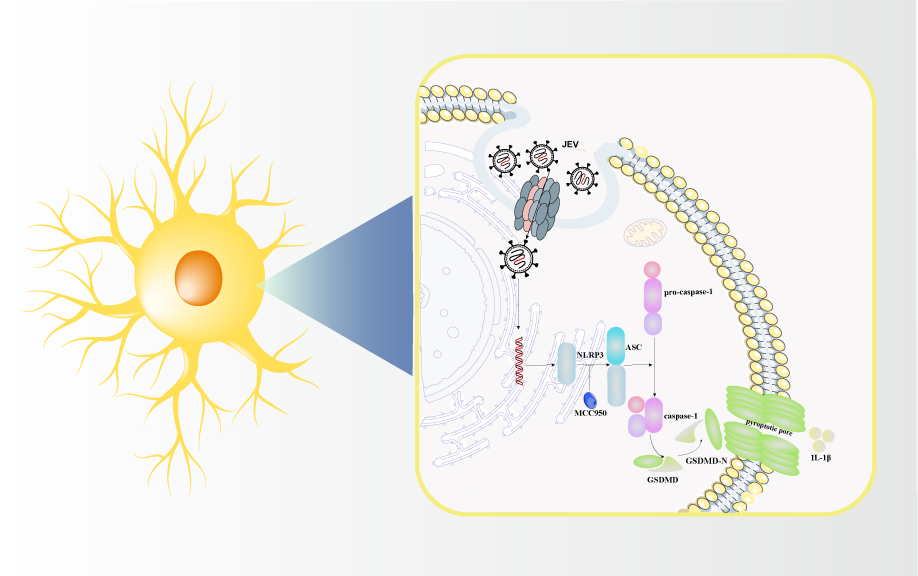

Supplement: S1 Fig — (TIF) [file pntd.0013924.s002.tif]
