# Supplementary material for: Japanese encephalitis virus activates the NLRP3/caspase-1/GSDMD signaling pathway in dopaminergic neurons
Source: PLoS Negl Trop Dis. 2026 Jan 22;20(1):e0013924. doi: 10.1371/journal.pntd.0013924 (PMC12893654; doi:10.1371/journal.pntd.0013924)
Supplement: Attachment — Submitted filename: Response.docx [file pntd.0013924.s003.docx]

**Reviewers' Comments:**

Reviewer's Responses to Questions

**Key Review Criteria Required for Acceptance?**

As you describe the new analyses required for acceptance, please consider the following:

**Methods**

-Are the objectives of the study clearly articulated with a clear testable hypothesis stated?

-Is the study design appropriate to address the stated objectives?

-Is the population clearly described and appropriate for the hypothesis being tested?

-Is the sample size sufficient to ensure adequate power to address the hypothesis being tested?

-Were correct statistical analysis used to support conclusions?

-Are there concerns about ethical or regulatory requirements being met?

Reviewer #1: (No Response)

Reviewer #2: The objectives of the study are clearly articulated in last paragraph of the introduction section, an appropriate study design, and statistical analyses were implemented. A clear ethical statement was also provided.

**Results**

-Does the analysis presented match the analysis plan?

-Are the results clearly and completely presented?

-Are the figures (Tables, Images) of sufficient quality for clarity?

Reviewer #1: (No Response)

Reviewer #2: Overall results are presented clearly and completely.

A few minor suggestions:

Line 116: Please provide the full term for this abbreviation FRET/FCCS.

Figure 1 C and D: The color intensity between 3 dpi and 6 dpi is a bit hard to interpret. Maybe use a more distinct variation? This goes for all the similar graphs throughout the manuscript.

Response: Thank you for your valuable comments.

Regarding the full names of FRET/FCCS, we have supplemented the information in the manuscript, with specific details provided on lines 112-113. The full designations are Förster-type resonance energy transfer (FRET) combined with fluorescence cross-correlation spectroscopy (FCCS).

We highly appreciate your inquiry about the immunofluorescence staining section. Indirect immunofluorescence staining was employed to label the target proteins, and detailed information on the primary antibodies and fluorescent secondary antibodies is available in the "Antibodies" subsection of the Materials and Methods section (please refer to corresponding lines 591-592, lines 607-611 for specifics). For the two potentially interacting proteins, we selected anti-rabbit IgG (H+L)-FITC (AS001, ABclonal, China) and sheep anti-mouse IgG (H+L)-Cy3 (AS007, ABclonal, China) as fluorescent secondary antibodies to bind with their respective primary antibodies, thereby localizing the target proteins. Both dyes have an excitation wavelength of 488 nm, which may pose a potential crosstalk risk. However, their emission wavelengths are 525 nm (FITC) and 570 nm (Cy3), and the corresponding detection channels do not overlap. The fluorescent microscope used in this study effectively mitigates the risk of crosstalk, supporting the selection of FITC and Cy3 for labeling. In subsequent research, the research team will further optimize the selection of fluorescent dyes to improve detection accuracy.

**Conclusions**

-Are the conclusions supported by the data presented?

-Are the limitations of analysis clearly described?

-Do the authors discuss how these data can be helpful to advance our understanding of the topic under study?

-Is public health relevance addressed?

Reviewer #1: (No Response)

Reviewer #2: The conclusions are supported by the presented data.

Would have been better if some of the study limitations were mentioned.

Also suggest including a final sentence that ties everything together and ultimately emphasizes how these findings are relevant to public health.

Response: Thank you for your valuable comments.

We have added a discussion on the limitations of the current study at the end of the Discussion section. One key limitation is that the cell model employed cannot fully recapitulate the in *vivo* physiological context (see corresponding lines 494-503). Following your suggestions, we have further elaborated on additional limitations (see corresponding lines 504-516): in this study, we focused solely on neurons primarily responsible for neurotransmitter secretion, neglecting the cross-reactions among neurotransmitters. While we attempted to explore the potential pathological responses of microglia to dopaminergic neuron damage, our investigation was confined to the NLRP3/ASC/GSDMD signaling pathway, lacking more direct evidence to substantiate the interaction between microglia and neurons.

Regarding the question of "how these findings are relevant to public health," we have supplemented the corresponding discussion in the manuscript. For specific details, please refer to line 511-516. The following is the specific content. Accordingly, in our subsequent research, we will prioritize identifying the key sites and temporal windows where microglia exert their effects during viral neuronal infection. This work aims to provide critical evidence for the development of inhibitors targeting excessive microglial activation and neuronal inflammation. Furthermore, these inhibitors are expected to offer novel insights into the prevention and control of Japanese encephalitis virus and other mosquito-borne diseases, guiding the rational allocation of public health resources, facilitating the formulation of personalized protection plans for high-risk populations prone to inflammatory storms, improving prevention efficacy, and reducing the associated medical burden.

**Editorial and Data Presentation Modifications?**

Use this section for editorial suggestions as well as relatively minor modifications of existing data that would enhance clarity. If the only modifications needed are minor and/or editorial, you may wish to recommend “Minor Revision” or “Accept”.

Reviewer #1: (No Response)

Reviewer #2: Line 44: Would be great if a reference/s could be provided to back the number of reported cases worldwide.

Response: Thank you for your valuable comments.

We have emphasized the hazards of Japanese encephalitis in the Introduction section, with specific details as follows in lines 41-50: Studies indicate that over 2 billion people reside in endemic regions worldwide, with approximately 50,000–175,000 reported cases of Japanese encephalitis virus infection annually. The case fatality rate ranges from 20% to 30%, while 30% to 50% of infected individuals suffer from neurological sequelae.

These data are sourced from the World Health Organization (WHO), and the corresponding fact sheet is accessible via the following link: [https://www.who.int/zh/news-room/fact-sheets/detail/japanese-encephalitis](https://www.who.int/zh/news-room/fact-sheets/detail/japanese-encephalitis). The latest update date of this document is August 6, 2024.

**Summary and General Comments**

Use this section to provide overall comments, discuss strengths/weaknesses of the study, novelty, significance, general execution and scholarship. You may also include additional comments for the author, including concerns about dual publication, research ethics, or publication ethics. If requesting major revision, please articulate the new experiments that are needed.

Reviewer #1: In this manuscript, the authors have investigated both in vivo and in vitro NLRP3 inflammasome activation in neurons following JEV infection. While this study has some interests, I have major criticisms:

-Figure 2C. In this figure, is it caspase-1 or activated caspase-1 that is detected in IF? Likewise, is it GSDMD or cleaved GSDMD that is detected? Indeed the authors wrote line 164 “Collectively, these findings demonstrate that the NLRP3 inflammasome mediates classical pyroptosis in JEV-infected dopaminergic neurons, linking Caspase-1 activation and GSDMD upregulation to neuroinflammatory pathogenesis.” Actually, caspase-1 (pro-caspase-1) and GSDMD upregulation is not a marker of NLRP3 inflammasome activation per se, since caspase-1 has to be matured through self-processing to process GSDMD into its pro-pyroptotic form and to cleave pro-Il1b and pro-IL-18 into their active form.

Response:Thank you for your valuable comments.Thank you for your valuable comments.

The proteins identified by IF are pro-caspase-1 and GSDMD-FL. Our research focus lies in whether neurons in brain tissue highly express these genes. As existing studies have shown, the basal expression levels of these genes in brain tissue are much lower than those in immune organs such as the spleen. Their expression is mainly restricted to specific cell types and can be significantly upregulated under neurological diseases or inflammatory conditions. Through IF, we observed that their expression levels increased significantly following viral infection, suggesting that viral infection activates the related pathways. Subsequently, we detected their precursors and cleaved forms using Western blot, which validated our hypothesis and confirmed its rationality.

Regarding the description in line 164, we have rephrased it as follows in lines 159-164: "The above results indicate that neural cells, represented by dopaminergic neurons, accumulated key executive proteins of pyroptosis before and after JEV infection, suggesting their potential capacity to undergo pyroptosis. Taken together, after viral infection, the NLRP3 inflammasome may be undergoing assembly; with inflammasome activation, this further suggests that pyroptosis may be initiated during this process."

-In figure 3, qPCR and WB were performed on mock-infected and JEV-infected mouse brains where other cells (glial and microglial cells) than neurons are present so that the presented results may be not specific of neurons… While during NLRP3 inflammasome activation, it is well documented that NLRP3 and pro-IL1B expression is increased following the priming as a consequence of NF-kB activation, the transcriptional increase of ASC, caspase-1 or GSDMD is known to be much less marked than for NLRP3 and pro-IL-1β. I’m therefore surprised to observe here such an increase in ASC expression and GSDMD following infection. In contrast and surprisingly, increase in pro-IL-1b expression is rather week.

Response: Thank you for your valuable comments.

For the study presented in Figure 3, our objective was to further validate a hypothesis derived from our research group’s prior studies: JEV infection in mouse brain tissue induces activation of the canonical pyroptosis pathway centered on NLRP3. The experimental results were consistent with transcriptome sequencing data from our group’s earlier work. Building on this, we raised a new question: Can neurons independently trigger pyroptosis, in contrast to immune cells. To explore this aspect, the above-mentioned research on neuronal cell pyroptosis was conducted.

Regarding your concerns about the increased expression of ASC, GSDMD and pro-IL-1β, we have found preliminary support in existing literature. Similar to macrophages, brain-resident immune cells such as microglia may regulate NF-κB activity, thereby inhibiting transcriptional upregulation of NLRP3 and pro-IL-1β. Notably, ASC contributes to NLRP3 inflammasome assembly and subsequent activation by forming dimers and/or oligomers localized to the detergent-insoluble cellular fraction—this process may not be strictly sequential with NF-κB activation. Earlier studies have even suggested that ASC can disrupt the interaction between RIP2 and caspase-1, redirecting caspase-1 from RIP2-mediated NF-κB activation toward pro-IL-1β processing. Additionally, GSDMD is thought to be expressed prior to the activation of NF-κB-related signaling pathways. While some viral infection studies indicate that IL-1β production requires certain factors, it can also be generated independently of ASC or caspase-1.

In our observations, ASC and GSDMD expression increased significantly in mouse brain tissue, whereas IL-1β upregulation was relatively modest. This may be attributed to the presence of non-neuronal immune cells in brain tissue: during early infection, resident immune cells (e.g., microglia) are rapidly activated and preferentially upregulate core inflammasome components (ASC) and cell death executors (GSDMD) to prepare for the rapid clearance of infected cells. However, to avoid excessive tissue damage, the body strictly restricts systemic expression of the potent proinflammatory cytokine pro-IL-1β via robust intracellular negative feedback mechanisms. Nevertheless, such feedback cannot fully control persistent viral infection or ongoing cell death. Thus, the marked upregulation observed in tissue homogenates reflects a balance between a "rapidly activated cell death program" and "strictly regulated inflammatory factor production."For the above discussion, we have touched upon lines 484-493.

To address these findings, in the future, we will employ cell-type-specific isolation (e.g., flow cytometry sorting) and spatial transcriptomics to precisely investigate whether ASC/GSDMD and pro-IL-1β are expressed in distinct cell populations, thereby directly validating our "cooperative division" hypothesis.

The above references can be found below:

Li Z, Wang Y, Huang W, Shi X, Ma T, et al. (2024) miR-155 induces sepsis-associated damage to the intestinal mucosal barrier via sirtuin 1/nuclear factor-κB-mediated intestinal pyroptosis. Acta biochimica et biophysica Sinica 56: 1618-1632.

Sarkar A, Duncan M, Hart J, Hertlein E, Guttridge DC, et al. (2006) ASC directs NF-kappaB activation by regulating receptor interacting protein-2 (RIP2) caspase-1 interactions. Journal of immunology (Baltimore, Md. : 1950) 176: 4979-4986.

Fan X, Han J, Zhong L, Zheng W, Shao R, et al. (2024) Macrophage-Derived GSDMD Plays an Essential Role in Atherosclerosis and Cross Talk Between Macrophages via the Mitochondria-STING-IRF3/NF-κB Axis. Arteriosclerosis, thrombosis, and vascular biology 44: 1365-1378.

Luo Y, Xiong B, Liu H, Chen Z, Huang H, et al. (2020) Koumine Suppresses IL-1β Secretion and Attenuates Inflammation Associated With Blocking ROS/NF-κB/NLRP3 Axis in Macrophages. Frontiers in pharmacology 11: 622074.

Suwanmanee S, Ghimire S, Edwards J, Griffin DE (2023) Infection of Pro- and Anti-Inflammatory Macrophages by Wild Type and Vaccine Strains of Measles Virus: NLRP3 Inflammasome Activation Independent of Virus Production. Viruses, 15(2), 260.

-In figure 5, like in figure 2C, which form of caspase-1 and GSDMD is detected? During NLRP3 inflammasome activation, ASC has been shown to form “specks” but here, it does not seem to be the case. Why?

Response:Thank you for your valuable comments.

In this study, we continued to examine pro-caspase-1 and GSDMD-FL. Regarding the characteristic speckle structure of ASC you mentioned, its observation generally requires a high-resolution confocal microscope equipped with a high-magnification objective. Unfortunately, the ordinary inverted fluorescence microscope used in our study lacks such high resolution, precluding the visualization of clear speckles.

However, as noted, the expression level of ASC in MN9D neurons is extremely low, which avoids interference from endogenously produced ASC in our research. Our aim was to validate the hypothesis—via immunofluorescence staining—that viral infection of neurons triggers an inflammatory response intrinsic to the neurons themselves. The results supported this: uninfected cells exhibited no obvious ASC-positive signals, whereas infected cells showed substantial ASC expression.

References for similar methodological approaches are provided as follows:

Ding S, Yan H, Gao J, Chen Y, Zhang N, et al. (2024) Genetic deletion of the apoptosis associated speck like protein containing a card in LysM+ macrophages attenuates spinal cord injury by regulating M1/M2 polarization through ASC-dependent inflammasome signaling axis. Experimental Neurology 382: 114982.

Tang X, Liu X, Wang Z, Chen M, Zhang D (2023) Molecular Characterization, Expression, and Regulatory Signal Pathway Analysis of Inflammasome Component Apoptosis-Associated Speck-like Protein Containing a CARD Domain (ASC) in Large Yellow Croaker (Larimichthys crocea). International journal of molecular sciences, 24(3), 2175.

-In figure 6, secretion of IL-1b and IL-18 should be assessed in JEV-infected MN9D cells by ELISA.

Response:Thank you for your valuable comments.

After preliminary experiments, we found that the cell supernatant containing high-titer viruses would cause severe technical interference in ELISA tests, resulting in abnormally elevated background signals and making it impossible to obtain reliable and valid data. We speculate that this might be due to the non-specific binding of virus particles to the microplate or the detection reagents. In this case, we cannot provide valid experimental results. More importantly, we believe that the Western Blot data we have obtained provides more mechanism-informed evidence for pyroptosis activation than ELISA. ELISA is mainly used for quantifying total secretion levels, while Western Blot can directly show the protein hydrolysis cutting process of caspase-1 on pro-IL-1β, which is the decisive step in the activation of the pyroptosis pathway. The above data are presented in Figure 6. Additionally, this discovery is corroborated by the data we provided such as ASC spot formation, Caspase-1 cleavage bodies formation, and GSDMD cleavage body formation, forming a complete evidence system that strongly supports our conclusion. Therefore, we believe that the data we currently provide is sufficient and rigorous enough to prove that viral infection can effectively induce cell pyroptosis and the maturation of related cytokines. We hope that in the reviewers' opinion, these in-depth mechanism data can make up for the absence of ELISA data."

-Figure 6A. NLRP3 activation leads to the recruitment of the adaptor ASC and pro-caspase-1 to form the inflammatory complex. However, their basal expression (amount of protein produced) is not necessarily directly increased by NLRP3 activation. What changes is their assembly into specks (ASC specks) and the cleavage/activation of caspase-1 (switch from pro-caspase-1 to active caspase-1 p20/p10). Upon inflammasome activation, caspase-1 cleaves GSDMD into its N-terminal fragment, which forms membrane pores and triggers pyroptosis. Here too, it is not necessarily the total expression of GSDMD that increases, but its cleavage into the active form (GSDMD-N).

Hence, again, the fact that JEV infection increases expression of NLRP3, ASC, caspase-1, GSDMD, pro-IL-1b/-18 is not a formal marker for NLRP3 inflammasome activation, rather a marker for NF-kB activation (likely as consequence of TLRs or RLRs stimulation) as confirmed by the level of TNF-a.

Response:Thank you for your valuable comments.

Regarding your questions, our previous research has confirmed that JEV infection significantly activates the NF-κB pathway, and this part of the experimental results has been published. The present study focuses more on the cascade signaling pathway centered on the NLRP3 inflammasome, with particular attention to the components of the NLRP3 inflammasome (NLRP3, ASC, caspase-1), the downstream key protein GSDMD, and the expression of the inflammatory factor IL-1β.

On one hand, JEV-induced upregulation of components such as NLRP3 not only indicates activation of the NF-κB pathway but also provides a molecular basis for potential inflammasome assembly. On the other hand, to confirm that the inflammasome is indeed activated, we further detected its functional markers, including the effective cleavage of caspase-1, GSDMD, and IL-1β, as well as the effective expression of ASC. These results were verified by IFA and WB, confirming that JEV does activate the NLRP3 inflammasome and trigger pyroptosis.

The relevant references are as follows:

Gao M, Liu Z, Guo X, Zhang J, Cheng G, et al. (2023) Japanese encephalitis virus induces apoptosis by activating the RIG-1 signaling pathway. Archives of virology 168: 169.

-Figure 6B. WBs are really poor quality. Cleaved GSDMD and IL-1b are detected 12hrs post-infection, not after 24h but detected again after 36hrs, this is not logical. Likewise, there are variations in the expression of NLRP3, ASC and GSDMD that are difficult to explain.

Response: Thank you for your valuable comments.

We acknowledge the technical challenges in Western blot analysis, which stem primarily from inherent difficulties associated with pyroptosis: membrane rupture leads to protease leakage, and even in the presence of inhibitors, samples remain at risk of degradation during processing. Additionally, variations in the extent of cell death across different time points complicate accurate protein quantification and consistent loading.

Regarding the expression trends of GSDMD and IL-1β, we attribute these patterns to the asynchrony of viral infection within the cell population. A subpopulation of "rapid responder" cells drives the initial response (12 hours), followed by a delayed response (36 hours) dominated by cells with slower infection progression. The 24-hour window represents a transitional phase between the completion of pyroptosis in the first wave of responder cells and the peak of the second wave. Such asynchrony is reasonable and common in virological studies, particularly in models using non-synchronized infected cell lines. As supported by previous literature, this phenomenon may arise because a small subset of cells is synchronously infected with high viral loads at the onset. These cells rapidly complete the viral replication cycle, reaching a threshold of PAMPs (e.g., dsRNA) to trigger the first round of robust inflammasome activation. Subsequently, the first wave of "rapid responders" undergoes pyroptosis and is cleared between 12–24 hours. In contrast, other infected but slower cells ("slow responders") have not yet accumulated sufficient viral replication or signaling molecules to reach the activation threshold, leaving the culture system in a state where "the first wave has perished while the second wave has not yet initiated." Shortly thereafter, the "slow responders" reach peak viral load, triggering a second round of inflammasome activation. ATP, DAMPs, or low levels of mature IL-1β released by the first wave may act as "alarm signals," priming surrounding slow responders in an autocrine/paracrine manner to enhance their sensitivity to virus-induced activation signals, thereby triggering a more synchronized burst. These time points likely correspond to 12h, 24h, and 36h; beyond this, as viral infection continues to spread, GSDMD-N and IL-1β expression increases progressively, resulting in significant differences. Similar mechanisms underlie the expression patterns of NLRP3, ASC, and GSDMD-FL, reflecting dynamic host-virus interactions in our model.

These data reveal a complex scenario: the virus undergoes multiple rounds of replication and spread within the cell population, with each round triggering a robust innate immune response that subsequently subsides as responding cells undergo pyroptosis. In a simplified, synchronized in vitro system, one might expect a single peak; however, in our model of JEV-infected neurons, the asynchrony of infection and multiple rounds of interplay between viral replication, cell death, and host feedback collectively shape this complex expression profile. The significant upregulation observed at 60 hours post-infection may reflect pathway hyperactivation or failure of feedback mechanisms under conditions of extremely high viral load in the culture system. This also highlights new directions for future research, such as using single-cell RNA sequencing to identify distinct responder subpopulations or employing viral replication inhibitors to interrupt this cycle, thereby facilitating the screening of inhibitors that mitigate pyroptosis-induced neuronal damage. Based on these results, we propose that the fluctuating expression of NLRP3 and related proteins reflects a dynamic balance between NF-κB-driven synthesis and caspase-1-driven consumption. Importantly, the detection of cleaved GSDMD and IL-1β is highly specific, and their biphasic appearance is consistent across biological replicates, providing robust support for our conclusion that JEV induces multiphase pyroptosis in neurons. We have revised this part again. For details, please refer to lines 278-297.

The potential mechanisms mentioned above are referenced in the following literature:

Latz E, Xiao TS, Stutz A (2013) Activation and regulation of the inflammasomes. Nature reviews. Immunology 13: 397-411.

Lu A, Magupalli VG, Ruan J, Yin Q, Atianand MK, et al. (2014) Unified polymerization mechanism for the assembly of ASC-dependent inflammasomes. Cell 156: 1193-1206.

de Vasconcelos NM, Van Opdenbosch N, Van Gorp H, Parthoens E, Lamkanfi M (2019) Single-cell analysis of pyroptosis dynamics reveals conserved GSDMD-mediated subcellular events that precede plasma membrane rupture. Cell death and differentiation 26: 146-161.

Place DE, Kanneganti T (2020) The innate immune system and cell death in autoinflammatory and autoimmune disease. Current opinion in immunology 67: 95-105.

Almeida-da-Silva CLC, Moreira-Souza ACDA, Ojcius DM (2024) Traditional approaches and recent tools for studying inflammasome activity. Journal of immunological methods 533: 113744.

-Figure 7. MCC950 is a specific inhibitor of NLRP3 inflammasome by preventing conformational activation of NLRP3. It Inhibits the formation of the NLRP3-ASC-caspase-1 complex reducing caspase-1 cleavage, thus the maturation and secretion of IL-1β and IL-18, as well as GSDMD-dependent pyroptosis. Hence, I do not understand why MCC950 inhibits expression of NLRP3, ASC, procaspase-1, GSDMD as observed by qPCR and WB. In your hands, MCC950 seems to act like a NF-kB inhibitor… this is so puzzling.

Response:We greatly appreciate this insightful comment from your opinion. For this part of the research, we have provided additional information in the discussion section. For details, please refer to line 456-466.

We have also observed that MCC950 not only inhibits NLRP3 activity (as evidenced by reduced caspase-1 cleavage and IL-1β maturation) but also unexpectedly decreases the transcriptional and protein levels of NLRP3, ASC, pro-caspase-1, and GSDMD. At first glance, this phenomenon appears inconsistent with the classical mechanism of MCC950, but we believe it reveals a deeper biological principle.

Our central hypothesis is as follows: In JEV-infected neurons, the activated products of the NLRP3 inflammasome (e.g., mature IL-1β/IL-18) and DAMPs released by pyroptotic cells collectively form a robust positive feedback loop, which in turn profoundly promotes the expression of inflammasome components. When MCC950 potently inhibits NLRP3 activation, it simultaneously disrupts this self-amplifying signaling loop. Thus, the observed reduction in expression is not a direct result of MCC950 inhibiting NF-κB, but rather an indirect consequence of its successful blockade of NLRP3 activity, which eliminates a strong NLRP3-dependent positive feedback signal. This model is supported by a growing body of recent literature, which indicates that inflammasome activity is tightly linked to the sustained expression of inflammatory genes. In our experimental context, JEV provides a persistent and intense stimulus, making this positive feedback effect particularly prominent. Notably, despite the significant inhibition of NLRP3 inflammasome-related components, pathological damage was not significantly attenuated, and microglia still exhibited a marked immune response. This suggests that the inhibitor used cannot fully abrogate the perpetual immune response in the host, highlighting the need for future research to target the excessive activation of microglia.

In summary, our data not only confirm the efficacy of MCC950 as an NLRP3 inhibitor but also reveal a new dimension of its action in complex infection models: by interrupting the self-amplifying cycle of inflammatory signals, it fundamentally reduces the overall level of the inflammatory network. Concurrently, these findings indirectly indicate that while a single inhibitor can block responses in related pathways, addressing pathological damage caused by viral infections requires further exploration into strategies that block viral replication and invasion. The above content has been emphasized in the discussion section. For details, please refer to lines 465-475.

The potential mechanisms mentioned above are referenced in the following literature:

Swanson KV, Deng M, Ting JP (2019) The NLRP3 inflammasome: molecular activation and regulation to therapeutics. Nature reviews. Immunology 19: 477-489.

Ferrari D, Casciano F, Secchiero P, Reali E (2021) Purinergic Signaling and Inflammasome Activation in Psoriasis Pathogenesis. International journal of molecular sciences, 22(17), 9449.

Vora SM, Lieberman J, Wu H (2021) Inflammasome activation at the crux of severe COVID-19. Nature reviews. Immunology 21: 694-703.

Paik S, Kim JK, Shin HJ, Park E, Kim IS, et al. (2025) Updated insights into the molecular networks for NLRP3 inflammasome activation. Cellular & molecular immunology 22: 563-596.

-WBs in Figure 8E are not convincing.

Response:Thank you for your valuable comments.We have revised it. For details, please see lines 395-410. In the present study, we observed that following JEV infection of microglia, the components of the NLRP3 inflammasome exhibited a trend of decreasing first and then increasing, with no statistically significant differences. This finding is consistent with our research group’s previous work, which demonstrated that microglia undergo significant apoptosis upon JEV infection.

Minor comments:

Do dopaminergic neurons produce IL-1b or IL-18?

Response: Thank you for your valuable comments.

In previous literature, we have observed that dopaminergic neurons typically do not express pro-IL-1β, or express it only at extremely low levels. Under pathological conditions such as Parkinson's disease or inflammatory stimuli, neurons can upregulate pro-IL-1β synthesis via activating the NF-κB signaling pathway upon receiving stimuli from activated microglia or intrinsic stress signals. This can be regarded as preparing "molecular substrates" for the inflammatory response. However, during the activation phase, dopaminergic neurons generally lack an efficient and complete mechanism for inflammasome assembly and caspase-1 activation. Therefore, despite their ability to synthesize pro-IL-1β, they often fail to effectively process and secrete it as mature IL-1β. The primary source of mature IL-1β in the brain is microglia, a type of professional immune cell. Dopaminergic neurons act more as "participants" and "amplifiers" of inflammatory signals rather than primary executors.

In contrast, the relationship between dopaminergic neurons and IL-18 is more direct and autonomous. Even under basal conditions, they continuously express the precursor of IL-18 (pro-IL-18), and its expression can be further upregulated upon pathological stimulation. More critically, studies have shown that dopaminergic neurons possess a complete molecular machinery required for IL-18 activation, including inflammasome components and caspase-1. This means they can independently process pro-IL-18 into mature, biologically active IL-18 and release it.

The relevant references are as follows:

Sugama S, Wirz SA, Barr AM, Conti B, Bartfai T, et al. (2004) Interleukin-18 null mice show diminished microglial activation and reduced dopaminergic neuron loss following acute 1-methyl-4-phenyl-1,2,3,6-tetrahydropyridine treatment. Neuroscience 128: 451-458.

Pike AF, Varanita T, Herrebout MAC, Plug BC, Kole J, et al. (2021) α-Synuclein evokes NLRP3 inflammasome-mediated IL-1β secretion from primary human microglia. Glia 69: 1413-1428.

Line 37, I guess that the authors meant « Pyroptosis » rather than “necroptosis”.

Response:Thank you for your valuable comments. We have made revisions in the corresponding parts of the article. For details, please refer to lines 35.

Reviewer #2: Overall, this is a well-written paper presenting exciting and impactful findings.

Wishing the researchers the very best moving forward.

Response: Thank you sincerely for your positive and encouraging feedback on our paper—it means a great deal to the team to have our work recognized in this way. We deeply value your well wishes and will carry this momentum as we advance follow-up work.
